# Supplementary material for: Chromothripsis during telomere crisis is independent of NHEJ, and consistent with a replicative origin
Source: Genome Res. 2019 May;29(5):737–49. doi: 10.1101/gr.240705.118 (PMC6499312; doi:10.1101/gr.240705.118)
Supplement: Supplemental Material [file supp_gr.240705.118_Supplemental_file_1.zip › contigs/annotated_contigs/DB38/contig.2.DB38_length_930_mean_cov_8.86559139785.docx]

**DB38_length_930_mean_cov_8.86559139785**

```
TGCAGTAGACAAAGAAAGAGGAAGGCAAGGAATACTGGAACCAGGAGCTTCACAGGATAGTGGGAAAAGGAAGCCTAGGATGAAAGTGA
 >chr2:46320996-46321435 - E=4e-246
AGTGGGATGCCAGGGTGACAGCTTTGCAGCAGGCACACAGGGCAGAAGGTAACCATTTAGACTGGAATAGTTTACAAGGCTTGGCAAAG

AATGTATCCAAGAATATGAAATTGATAGAATACTGCTTATACCTAAACATGCTAAAAGGAGATATGGACAGTTTGTAGAGAGTTTGTGG

TTGAATCAATAATAATTACATAGAAAACAAAGAGATATTTATTAACTTCAGGGAAAATAAACAAAAAAAATGCACTGGGAAGGAAAAGT

AACTATAGCATACCACATAACTGAATTCCAAATGGTATTTCACAGTTATAATAATGTATACTATATATTCATTTAACCAAAA|T|GAGG
                                                                                   >chr
TGGGAGGATCACTTGAGTCCAGGACTTCGAGACCAGCCTGGGCAACATGGTAAGACCCTGTCTCTTAAAAAAATTAGCTGGGCATGGTG
2:46376732-46377241 - E=6e-263
GTACGTGTCTGTAGTCTCACCTTCTCAGAAGGCTGAGGTGGAAAAATCACTTGAGCCCGGGAGTTTGAGGCTGTAGTGAGTCATAATCA

TGCGACTGCACTTCAACCTGGGTAACAGAGTGAGACCCTATCTCAAAAAAAAAAAAGGATCTTTTCTGATTGGCTATTCTATTTGATCT

GCTCTCCATACACATATGTCATAAATACATGATACACACACACGCACATATAAATATGTAGCATGTCAATGACATATATATATAAATAC

ATATATATAAAAATACATATATATATATATGGAGATGACATCCAATAGAAAAGACTAGAATTTAATATCACCTTTCAGCTGTATAGAGC

TTTGTATTTTACAGTATGCCTTTAAAATATAACATATATAAA
```
